# Effects of Clinical Training and Case Difficulty on the Radiographic Quality of Root Canal Fillings Performed by Dental Students in Saudi Arabia

**DOI:** 10.7508/iej.2015.04.012

**Published:** 2015

**Authors:** Reem Siraj Alsulaimani, Kholod Khalil Al-Manei, Sara A. Alsubait, Razan Shafik AlAqeely, Sharifa A. M. Al-Shehri, Ebtissam M. Al-Madi

**Affiliations:** a * Department of Restorative Dental Science, King Saud University, Riyadh, Saudi Arabia; *; b *Department of Periodontics and Community Dentistry, King Saud University, Riyadh, Saudi Arabia; *; c *Department of Prosthetic Dental Science, King Saud University, Riyadh, Saudi Arabia*

**Keywords:** Obturation Density, Obturation Length, Preparation Taper, Procedural Accident, Root Canal Treatment

## Abstract

**Introduction:**

The aim of this study was to evaluate the effects of training duration and case difficulty on the radiographic quality of root canal fillings performed by dental students in Saudi Arabia.

**Methods and Materials:**

A longitudinal cohort study was conducted at King Saud University. Root canal treatments performed by 55 dental students from 2012-2014 were included in the study. Each student treated at least five teeth during the first year of clinical endodontic training and another five teeth during the second year. Case difficulty was assessed based on tooth position in the dental arch and preoperative conditions. The radiographic quality of the root canal filling was evaluated by two endodontists blinded to treatment completion date. The evaluation criteria were adequate obturation, presence of mishaps and preparation taper. The data were statistically analysed using univariate and multivariate logistic regression analyses; and the level of significance was set at 0.05.

**Results:**

Inadequate obturation and mishaps were significantly less prevalent in teeth treated after 2 years of clinical training. The odds ratios for inadequate obturation and mishaps increased significantly as tooth position moved posteriorly. Inadequate obturation and more mishaps were significantly more prevalent in teeth with preoperative conditions. Preparation taper was not significantly affected by training duration or case difficulty**. **

**Conclusion:**

The quality of root canal fillings performed by Saudi students was adversely affected by case difficulty. The radiographic quality of root canal fillings improved significantly after 2 years of clinical training. Preparation taper outcome is likely dependent on the preparation technique and instrument taper.

## Introduction

The major objectives of root canal treatment (RCT) are to remove irritants from the root canal system, fill the cleaned and shaped system adequately and prevent future recontamination of the sealed root canal system [1]. Successful periapical healing following a root canal procedure is strongly associated with adequate RCT [2, 3]. Radiographically, adequate RCT can be defined as the canal(s) being filled within 2 mm of the radiographic apex with a proper taper and without the presence of mishaps [4, 5]. Conducting adequate RCT while simultaneously avoiding Procedural Accidents is a challenging task for dental students. Therefore, preclinical and clinical endodontic training is frequently assessed and updated in the undergraduate curriculum [6, 7].

Having recognized the importance of periodic assessment of the endodontic curricula, the American Association of Endodontics (AAE) developed the AAE Endodontic Case Difficulty Assessment Form. The AAE form allows for the consideration of potential risk factors such as tooth position in the arch and preoperative conditions. Morphologic aberrations of the crown, root curvature and previous RCT are common preoperative conditions. The effect of tooth type on the technical quality of RCT is well documented [8-10]. However, previous studies have not investigated the effects of common preoperative conditions on the quality of RCTs performed by dental students [8, 11, 12].

Researchers have consistently used radiographic parameters to evaluate the quality of root canal fillings performed by dental students [8, 12-14]. The major findings of previous studies suggest a significant association between inadequate RCT and both tooth type and root curvature. However, there was no reported significant effect of training duration on the technical quality of RCT. The absence of a positive training duration effect on the technical quality of RCT is inconsistent with the objective of 2 years of endodontic clinical training. To objectively assess the benefits of a 2-year endodontic training course, a study in which the same students are observed throughout their endodontic training is required. However, such a study has not been previously reported [12, 15]. Therefore, the aim of this longitudinal study was to evaluate the effects of training duration, tooth position in the dental arch and preoperative conditions on the radiographic quality of root canal fillings performed by dental students in Saudi Arabia.

## Materials and Methods

***Study participants***

The study design was approved by the Ethical Committee of the College of Dentistry Research Centre (CDRC), King Saud University, Riyadh, Saudi Arabia. The study was conducted in full accordance with the World Medical Association Declaration of Helsinki (2008). Verbal informed consent was approved by the Ethical Committee of CDRC and obtained from participating students and patients. Fifty-five students registered in the Bachelor of Dental Science (BDS) program were enrolled in the study. The BDS program includes 1 year of preclinical training on natural teeth and 2 years of endodontic clinical training. The first and second years of endodontic clinical training were in the fourth and fifth (senior) years of the BDS program, respectively.

The successful completion of preclinical training was a prerequisite for student enrolment in the study. From 2012 to 2014, each student completed RCTs on at least five teeth during their first year of clinical endodontic training and another five teeth during their second year. Therefore, each participant treated a total of 10 teeth or more during the 2-year endodontic clinical program. Teeth treated by the same students were collected and arranged chronologically according to the treatment completion date.

***Endodontic treatment***

Endodontic treatment was always supervised by an endodontist, with an average staff-to-student ratio being 1:7. Briefly, the access cavity was produced using aseptic techniques with rubber dam isolation and temporary build up for badly broken teeth. Working length was determined with an electronic apex locator (Root ZX, J. Morita USA, Inc., Irvine, CA, USA) and was confirmed radiographically. Cleaning and shaping of the root canal system was performed by using step-back preparation technique with stainless steel K-files (Dentsply, Tulsa, OK, USA) and a 1% sodium hypochlorite irrigation solution. Root canal filling was performed using cold lateral condensation with ISO-standardized gutta-percha cones and AH-Plus sealer (Dentsply, Tulsa Dental, Tulsa, OK, USA). In all treated teeth, the master apical cone was not less than #30. 

***Radiographic evaluation criteria***

Teeth included in the study had preoperative, postoperative and working radiographs, which were taken using the parallel technique with Kodak^®^ Ultra-speed D films (Carestream Health, Inc., Rochester, NY, USA). Radiographs were mounted in cardboard slits to block ambient light from entering the illuminated viewing box (Star X-ray Illuminator; Star X-ray, Amityville, NY, USA) and examined under 2× magnification with a magnifier.

Using the AAE Endodontic Case Difficulty Assessment Form and Guidelines as a reference, root canal-treated teeth were categorized by difficulty according to tooth position in maxillary and mandibular arch (anterior and premolars, first molars, second and third molars) and by the presence of preoperative conditions such as morphologic aberrations of the crown (normal crown morphology or full coverage restoration), degree of root curvature according to Schneider method (slight <10^°^ and moderate 10-30^°^) [16] and previous root canal filling (no previous root canal filling or previous root canal filling without complications). Teeth with two or more preoperative conditions were grouped and compared with teeth with one or no preoperative condition. The criteria used to evaluate the quality of root canal fillings included obturation length, obturation density, preparation taper and presence of mishaps, as described previously [4]. In multi-rooted teeth, the radiographic evaluation was based on the root that had the poorest quality of canal filling. 

***Intra-examiner reliability***

Radiographic evaluation was performed by two endodontists blinded to the students’ names and treatment completion dates. To determine intra-examiner reliability, each examiner’s evaluation scores were compared with a set of 20 periapical radiographs. The duration between the first and second readings was 2 weeks. The first examiner evaluated preoperative conditions and preparation taper, while the second examiner evaluated obturation length, obturation density and mishaps.

***Statistical analysis***

The statistical analysis was performed using SAS software (version 9.3, SAS Institute Inc., Cary, NC, USA). Because observations from the same student were likely to be correlated, univariate and multivariate logistic regression analyses for repeated categorical response data were used to investigate relationships between outcome variables (*i.e*. obturation length and density, preparation taper and mishaps) and independent variables (*i.e*. training duration, tooth position in the arch, and preoperative conditions). The reference group was as follows: 2 years of clinical training, anterior/premolar teeth, and no preoperative condition. Generalized estimating equations were used to fit the logistic regression models for repeated categorical response data [16, 17]. A first-order autoregressive (AR-1) structure was used for the covariance matrix to model the correlation between two separate measurements from the same student; this structure assumes that two measurements which are closer together in time, are more correlated than measurements that are farther apart. It was assumed that observations from different students were independent.

**Table 1:** *Distributions of the evaluated teeth (*n*=692).*

**Variable**	**N (%)**
Training duration	
1 year	312 (45.09)
2 years	380 (54.91)
**Preoperative condition**	
None	176 (25.43)
Full coverage restoration (FCR)	55 (7.95)
Retreatment	78 (11.27)
Curved (10–30°)	250 (36.16)
FCR and retreatment	54 (7.80)
Curved and retreatment	41 (5.92)
Curved and FCR	18 (2.60)
Curved and FCR & retreatment	20 (2.89)
**Tooth position in the dental arch**	
Central incisor	48 (6.94)
Lateral incisor	80 (11.56)
Canine	117 (16.91)
1st premolar	103 (14.88)
2nd premolar	148 (21.39)
1st molar	132 (19.08)
2nd molar	63 (9.10)
3rd molar	1 (0.14)
**Obturation length**	
Adequate	589 (85.12)
Inadequate: short >2 mm	65 (9.39)
Inadequate: long <2 mm	32 (4.62)
Inadequate: long >2 mm	6 (0.87)
**Obturation density**	
Adequate	622 (89.88)
Inadequate	70 (10.12)
**Mishaps**	
None	527 (76.16)
Transportation	54 (7.80)
Ledge	55 (7.95)
Perforation	48 (6.94)
Separated instrument	4 (0.58)
Missed canals	4 (0.58)
**Preparation taper**	
Adequate	615 (88.87)
Inadequate	77 (11.13)

The univariate and multivariate logistic regression analysis was used to test the hypothesis that training duration, tooth position in the arch and preoperative conditions significantly affect the quality of root canal fillings. The level of significance was set at 0.05. Odds ratio estimates for the independent variables and corresponding 95% confidence intervals were also calculated.

## Results

In total, RCT was performed on 692 teeth by 55 students during 2 years of endodontic clinical training. Adequate obturation length and density were rated in 589 (85.12%) and 622 (89.99%) teeth, respectively. Mishaps were detected in 165 (23.84%) teeth, and the preparation taper was adequate in 615 (88.87%) teeth. Table 1 shows the distributions of teeth according to the evaluated parameters.

The kappa values for intra-examiner reliability were 0.9, 1.0, 0.5, 0.8, and 0.8 for preoperative condition, preparation taper, obturation length, obturation density and mishaps, respectively.

***The effect of training duration***

In the univariate analysis, inadequate obturation and mishaps were significantly less prevalent in teeth treated after 2 years of clinical training than in those treated during the first year of clinical training (Table 2). However, a significant training duration effect was not detected when preoperative conditions and tooth position in the dental arch were included in the multivariate analysis. Table 2 presents the lack of a significant training duration effect on preparation taper in the univariate and multivariate analyses. 

**Table 2:** Univariate and multivariate analyses of the effects of training duration (1 year *vs.* 2 years) on the quality of root canal treatment

	**Univariate analysis**		**Multivariate analysis**	
	**OR** **(95% CI)**	***P***-**value**	**OR** **(95% CI)**	***P***-**value**
**Obturation length **	0.54 (0.36, 0.80)	0.0061*	0.70 (0.41, 1.19)	0.1867
**Obturation density **	0.42 (0.23, 0.77)	0.0067*	0.62 (0.35, 1.10)	0.0992
**Mishaps**	0.56 (0.40, 0.80)	0.0023a	0.78 (0.48, 1.28)	0.3305
**Preparation taper**	0.60 (0.36, 1.02)	0.0723	0.66 (0.38, 1.15)	0.1578

a. Statistically significant at *P*<0.05; *P*-value based on the Wald chi-squared test; OR=odds ratio

**Table 3:** Univariate and multivariate analyses of the effects of tooth position in the dental arch on the quality of root canal treatment

	**Comparison group**	**Reference group**	**Univariate analysis**		**Multivariate analysis**	
			**OR** (95% CI)	***P***-**value**	**OR** (95% CI)	***P***-**value**
**Obturation length **	1^st^ molar	Anterior/Premolar	0.15 (0.10, 0.23)	<0.0001 ^a^	0.16 (0.10, 0.26)	<0.0001^ a^
	2^nd^ and 3^rd^ molar	Anterior/Premolar	0.27 (0.13, 0.57)		0.29 (0.14, 0.62)	
	2^nd^ and 3^rd^ molar	1^st^ molar	0.54 (0.25, 1.13)		0.55 (0.25, 1.19)	
**Obturation density **	1^st^ molar	Anterior/Premolar	0.30 (0.16, 0.54)	0.0037 ^a^	0.38 (0.19, 0.76)	0.0310^ a^
	2^nd^ and 3^rd^ molar	Anterior/Premolar	0.38 (0.18, 0.82)		0.43 (0.19, 0.99)	
	2^nd^ and 3^rd^ molar	1^st^ molar	0.77 (0.35, 1.68)		0.88 (0.40, 1.93)	
**Mishaps**	1^st^ molar	Anterior/Premolar	0.09 (0.06, 0.14)	<0.0001^ a^	0.10 (0.06, 0.16)	<0.0001^ a^
	2^nd^ and 3^rd^ molar	Anterior/Premolar	0.23 (0.13, 0.41)		0.25 (0.14, 0.46)	
	2^nd^ and 3^rd^ molar	1^st^ molar	0.40 (0.22, 0,72)		0.40 (0.21, 0.77)	
**Preparation taper **	1^st^ molar	Anterior/Premolar	0.58 (0.33, 1.02)	0.2482	0.65 (0.37, 1.15)	0.3964
	2^nd^ and 3^rd^ molar	Anterior/Premolar	0.85 (0.43, 1.68)		0.90 (0.45, 1.78)	
	2^nd^ and 3^rd^ molar	1^st^ molar	0.69 (0.32, 1.46)		0.72 (0.34, 1.53)	

**a**. Statistically significant at *P*<0.05; *P* value based on the Wald chi-squared test; OR=odds ratio

**Table 4:** Univariate and multivariate analyses of the effects of preoperative conditions on the quality of root canal treatment

	**Comparison group**	**Reference group**	**Univariate analysis**		**Multivariate analysis**	
			**OR** (95% CI)	***P***-**value**	**OR** (95% CI)	***P***-**value**
**Obturation length **	One condition	None	0.47 (0.28, 0.80)	<0.0066^ a^	0.98 (0.53, 1.80)	<0.3536
	Two or more	None	0.46 (0.23, 0.90)		0.57 (0.25, 1.27)	
	Two or more	One condition	1.02 (0.56, 1.87)		1.72 (0.83, 3.55)	
**Obturation density **	One condition	None	0.55 (0.27, 1.12)	0.0160^ a^	0.78 (0.34, 1.81)	0.2566
	Two or more	None	1.43 (0.44, 4.62)		1.53 (0.42, 5.55)	
	Two or more	One condition	0.38 (0.16, 0.94)		0.51 (0.20, 1.31)	
**Mishaps**	One condition	None	0.29 (0.19, 0.50)	<0.0001^ a^	0.61 (0.37, 0.99)	<0.0443^ a^
	Two or more	None	0.27 (0.13, 0.54)		0.32 (0.14, 0.75)	
	Two or more	One condition	1.10 (0.62, 1,94)		1.87 (0.97, 3.63)	
**Preparation taper **	One condition	None	0.89 (0.48, 1.65)	0.8605	1.01 (0.54, 1.90)	0.9978
	Two or more	None	1.06 (0.50, 2.24)		0.99 (0.47, 2.10)	
	Two or more	One condition	0.84 (0.42, 1.65)		1.03 (0.49, 2.14)	

**a.** Statistically significant at *P* 0.05; *P*-value based on the Wald chi-squared test; OR=odds ratio

***The effect of tooth position in the dental arch***

Univariate and multivariate analyses (Table 3) showed that obturation length, density, and mishaps were significantly affected by tooth position in the dental arch. In addition, the odds ratios for inadequate obturation and mishaps increased significantly as tooth position moved posteriorly. Table 3 demonstrates the absence of a significant tooth position effect on preparation taper.

***The effect of preoperative conditions***

Table 4 demonstrates the significant effect of preoperative conditions on obturation length, density, and mishaps in the univariate analysis. Furthermore, the odds ratios in Table 4 suggest that inadequate obturation length was more likely to occur in teeth with two or more preoperative conditions than in teeth with no preoperative condition. Mishaps were significantly more prevalent in teeth with preoperative conditions. However, preparation taper was not significantly affected by the presence of preoperative conditions.

## Discussion

The radiographic evaluation of root canal fillings is a standard practice because periapical radiographs provide good contrast, which facilitates a reliable RCT evaluation. In this study, the radiographic quality of root canal fillings improved significantly as students advanced in their clinical endodontic training. For example, adequate obturation and fewer mishaps were observed in cases treated in the second year of clinical training. This could be explained by the improved manual skills and clinical orientation of a dental student with exposure to a greater number of endodontic cases. However, previous studies reported no difference in the radiographic quality of root canal fillings when fourth and fifth year BDS students were compared [12, 15]. The difference between the findings observed in this study and in previous studies is likely caused by differences in study design. For example, this study followed the same students for 2 years and compared their progress chronologically, while previous studies depended on random samples.

To eliminate any potential bias that could be caused by case difficulty on the evaluation of obturation length, obturation density, and mishaps, one examiner evaluated preoperative radiographs separately. Preparation taper and obturation density were each affected by the other; therefore, to further reduce the bias, one examiner evaluated preparation taper and the other evaluated obturation density. The kappa values obtained for intra-examiner reliability ranged from moderate to almost perfect, which is a result comparable to those of previous studies [5, 12].

In this study, adequate root canal fillings were observed more frequently in anterior and premolar teeth than in molars. Teeth with normal coronal morphology and a slight curvature exhibited fewer mishaps than teeth with a full coverage restoration, previous root canal filling, and those with curved roots. Therefore, the radiographic quality of root canal fillings was improved in teeth that were less difficult to work on. This finding could be beneficial in developing undergraduate education curricula because the AAE Endodontic Case Difficulty Assessment Form will enable supervising faculty to select cases with minimal-to-moderate difficulty for dental students to perform at the beginning of their endodontic training, and then provide increasingly difficult cases as the students’ progress. Appropriately adjusting case difficulty for students during their endodontic training should result in improved performance, *i.e*. adequate root canal fillings with fewer mishaps.

In this study, tooth position in the dental arch was the most significant factor affecting the incidence of mishaps, obturation length, and density. This finding was in agreement with other studies [4, 7, 8, 11, 18, 19]. 

Consistent with Vukadinov *et al.* and previous studies [4, 8, 18], moderate root curvature (10-30^°^) significantly reduced the quality of root canal fillings and increased the occurrence of mishaps. Additionally, retreatment and/or full coverage restoration with root curvature increased the incidence of mishaps. Therefore, the presence of previous treatment or coronal coverage in addition to root curvature appears to reduce the radiographic quality of root canal fillings performed by students. This is a new finding, as previous studies did not investigate the effect of retreatment or full coverage restoration on the quality of root canal fillings in teeth with curved canals [4, 8, 18]. 

Er *et al.* and Rafeek *et al.* [18, 20], reported that preparation taper was significantly associated with tooth type and root curvature [4]. In this study, however, no significant association was detected between preparation taper and any independent variable. One explanation for this finding is that preparation taper is dependent on the root canal preparation technique and instrument taper. The root canal preparation technique remained the same during this longitudinal study; therefore, a significant difference in preparation taper was not detected.

This study focused on investigating the reported lack of improvement in the quality of RCTs performed by students while using the step-back technique in clinical training [12, 15]. Therefore, the present study was limited to root canal preparation using K-files. However, future studies should examine the effects of using nickel-titanium rotary files on RCT technical quality while considering training duration and case difficulty.

## Conclusion

In conclusion, the radiographic quality of root canal fillings performed by Saudi dental students decreased when the treated teeth were located more posteriorly in the dental arch. Additionally, the presence of a full coverage restoration, root curvature, and previous RCT increased the occurrence of mishaps. However, the radiographic quality of root canal fillings improved after 2 years of clinical training. Therefore, we recommend that dental students treat cases with minimal difficulty at the beginning of their clinical training, and treat more teeth overall.
